# Neural Stem Cell Extracellular Vesicles Disrupt Midline Shift Predictive Outcomes in Porcine Ischemic Stroke Model

**DOI:** 10.1007/s12975-019-00753-4

**Published:** 2019-12-06

**Authors:** Samantha E. Spellicy, Erin E. Kaiser, Michael M. Bowler, Brian J. Jurgielewicz, Robin L. Webb, Franklin D. West, Steven L. Stice

**Affiliations:** 1 Regenerative Bioscience Center, University of Georgia, Athens, GA 30602 USA; 2 Department of Animal and Dairy Science, University of Georgia, Athens, GA 30602 USA; 3 ArunA Biomedical, Athens, GA 30602 USA

**Keywords:** Stroke, Magnetic brain imaging, Swine, Extracellular vesicles, Neural stem cells, Neuroimaging

## Abstract

Magnetic resonance imaging (MRI) is a clinically relevant non-invasive imaging tool commonly utilized to assess stroke progression in real time. This study investigated the utility of MRI as a predictive measure of clinical and functional outcomes when a stroke intervention is withheld or provided, in order to identify biomarkers for stroke functional outcome under these conditions. Fifteen MRI and ninety functional parameters were measured in a middle cerebral artery occlusion (MCAO) porcine ischemic stroke model. Multiparametric analysis of correlations between MRI measurements and functional outcome was conducted. Acute axial and coronal midline shift (MLS) at 24 h post-stroke were associated with decreased survival and recovery measured by modified Rankin scale (mRS) and were significantly correlated with 52 measured acute (day 1 post) and chronic (day 84 post) gait and behavior impairments in non-treated stroked animals. These results suggest that MLS may be an important non-invasive biomarker that can be used to predict patient outcomes and prognosis as well as guide therapeutic intervention and rehabilitation in non-treated animals and potentially human patients that do not receive interventional treatments. Neural stem cell–derived extracellular vesicle (NSC EV) was a disruptive therapy because NSC EV administration post-stroke disrupted MLS correlations observed in non-treated stroked animals. MLS was not associated with survival and functional outcomes in NSC EV–treated animals. In contrast to untreated animals, NSC EVs improved stroked animal outcomes regardless of MLS severity.

**Electronic supplementary material:**

The online version of this article (10.1007/s12975-019-00753-4) contains supplementary material, which is available to authorized users.

## Introduction

Therapeutic development for ischemic stroke has previously focused on small molecules, with anti-thrombotic, thrombolytic, and anti-inflammatory mechanisms of action [1]. While approximately 4% of over 430 promising clinical trials for these small molecular therapeutics have reached world markets [2], ischemic stroke continues to remain a leading cause of death and long-term disability worldwide [3]. This translational disconnect, from promising preclinical studies to late-stage clinical trial failure, has originated from a number of factors including (1) an absence of predictive functional outcome biomarkers [4], (2) a limited pipeline of cell-based neurorestorative and neuroprotective therapeutics [5–8], and (3) a lack of models more representative of the human stroke condition [8–12]. Predictive parameters, once identified, would serve to better assess the efficacy of therapeutics as well as aid clinical decisions surrounding acute therapeutic treatment [13] and long-term rehabilitation planning. Additionally, they could serve to parse out subpopulations of patients with differential prognosis or response rates to novel therapeutic treatments.

Identifying acute parameters which are predictive of long-term functional stroke outcomes has significant implications for characterizing patient injury severity, prognosis, and rehabilitation planning as well as offering improved efficacy assessments of neuroprotectants in preclinical studies [14, 15]. Historically, lesion volume has been regarded as one of the most important and predictive acute measurements in stroke clinical trials [16–20]. In rodent studies, however, the relationship between lesion volume and functional outcome is not well established. Lesion volume has proven predictive of behavioral tests such as the corner test, while having no predictive value of neurological score or number of foot-faults in modified testing [21]. Due to the overwhelming translational disconnect between therapeutic efficacy in animal studies and outcome improvements in later clinical trials, there has been a call to investigate other potential parameters which are effective predictors along the entire therapeutic development pipeline [22]. For example, diffusion tensor imaging (DTI)–derived fractional anisotropy (FA) measures of pyramidal tract integrity of patients following stroke have recently been shown to be predictive of residual motor function. The predictive capacity of this measurement, however, is often limited to specific neuroanatomical locations [23], thus greatly decreasing the range of cases in which it is reliably predictive. Additionally, successful acquisition and measurement of these parameters rely on specific equipment and software, specialized technician training, and a combination of additional clinical information for their implementation as a reliable predictive model of recovery [24]. Here, an analysis of multiple magnetic resonance imaging (MRI) measurements, including T2W lesion volume, DTI-derived FA, and 10 others, was directly compared to identify their predictive capacity for functional outcome measures at acute and chronic time points. From this unbiased approach, the overall most predictive MRI parameter in a porcine middle cerebral artery occlusion (MCAO) model of stroke could be statistically identified and further analyzed by a recovery scale commonly used in human medicine.

While identification of improved MRI biomarkers of stroke recovery and outcomes is essential, improving the ability to detect novel therapeutic options for stroke patients is warranted. Development and implementation of novel neuroprotective therapeutics are necessary to lead to holistic improvements in clinical patient outcomes and rehabilitation [25]. Recent clinical trials have demonstrated the safety and efficacy of stem cell therapeutics in stroke patients with treatments leading to reduced lesion volumes and improved modified Rankin scale (mRS) and National Institutes of Health Stroke Scale (NIHSS) scores [26, 27]. Unfortunately, there are limitations in the use of stem cell therapies such as the time and cost needed for autologous cell expansion, intra- and inter-donor variability, rejection of allogeneic therapies [28], tumor formation, transplanted cell viability [28], and limited integration and retention of cells in the intended sites [28–30]. Established clinical efficacy of transplanted cells, even with limited integration, however, suggests the therapeutic efficacy of these stem cell therapies can be attributed in whole or part to secreted and paracrine factors, such as extracellular vesicles [28, 31]. Moving to these cell-free therapeutic systems can overcome some of these current limitations associated with cell therapies [32].

In this study, the therapeutic efficacy of neural stem cell–derived extracellular vesicles (NSC EVs) as a therapeutic was assessed in relation to identified MRI parameters. These nanosized vesicles, of neural stem cell origin, which are comprised of proteins, mRNAs, miRNAs, lipids, and other factors with therapeutic potential, were previously described by our group in rodent [33] and porcine [34] models of ischemic stroke. In this study, the identified MRI parameters provided insight into the specific therapeutic effects of NSC EVs on subpopulations of treatment recipients with more severe infarct distortions.

Lastly, identification of MRI imaging biomarkers and novel therapeutic effects should be assessed in increasingly translational models of stroke. Our research team has developed a large animal, porcine MCAO model of ischemic stroke [34]. This model simulates human central nervous system anatomy with respect to white matter content, complexity, and size [35]. In addition to recapitulating tissue degeneration following stroke, these cytoarchitectural similarities of the porcine model allow for acquisition of MRI sequences and metrics which extend beyond measures available in rodent models. For example, limited spatial resolution of MR images in the rodent brain has traditionally precluded the measurement of midline shift (MLS) from clinically relevant structures such as the septum pellucidum [36, 37] and necessitated measurement from the third ventricle [38]. Additionally, unlike the lissencephalic rodent brain, the gyrencephalic brains of pigs and humans have increased heterogeneity in blood flow due to collateral circulation and differences in gray and white matter composition that are uniquely affected by stroke, resulting in differential rates of penumbra evolution [39]. These neuroanatomical similarities allow for a more faithful representation of clinical stroke progression and therefore greater confidence in the translational potential of identified predictive MRI parameters.

The objective of this study was to identify specific, simple, and easy-to-measure MRI parameters which were predictive of a range of functional outcomes at chronic time points, as well as capture potential differences in porcine stroke recovery following therapeutic intervention. In a previous study [34], 65 gait, 25 behavior, and 15 MRI parameters were measured in a MCAO porcine model of stroke and assessed here in a retrospective multiparametric correlation analysis. Additional measures, such as MLS, were not previously determined. This study demonstrated that MLS was highly correlative with a number of gait and behavioral outcomes at days 1 and 84 post-MCAO, modified Rankin scale (mRS) scores from days 0 to 6 post-MCAO, and overall survival at 84 days post-MCAO in non-treated animals. Furthermore, NSC EV treatment disrupted the correlation between MLS and functional outcomes demonstrating a positive therapeutic effect. The high sensitivity of MLS allowed identification of subpopulations of MCAO animals with high midline shift (HMLS) displaying differential functional responses to NSC EV therapy, which were not identified utilizing traditional analytical approaches. These findings have important implications for the utility of MLS in preclinical and clinical settings as a biomarker for stroke recovery and further support that NSC EV treatment is a strong stroke therapeutic candidate.

## Materials and Methods

### Porcine Middle Cerebral Artery Occlusion

Middle cerebral artery occlusion (MCAO) was performed by a veterinary neurosurgeon on castrated male landrace pigs [35]. Animals were randomly assigned to either a neural stem cell–derived extracellular vesicle (NSC EV) treatment or non-treated group [34]. Briefly, NSC EVs or PBS was administered at 2, 14, and 24 h post-stroke. The NSCs that generated the NSC EV were an adherent culture of homogenous cells that are POU5F1-negative and NESTIN and SOX2-positive, determined using immunocytochemistry and are of normal karyotype. The NSCs are proliferative and generated NSC EVs were harvested from the spent culture media. The NSC EVs, themselves, were previously characterized by NanoSight analysis and were found to have a size peak of 66 nM and 110 nM [33], and have a consistent marker profiler by MACSPlex, expressing CD81, CD29, CD41b, and MCPS [34]. Initially, 14 animals were included in the study with 7 animals in each group. Due to the high mortality rate in the non-treated group, 2 more animals were added for a total of 9 non-treated animals, 7 NSC EV–treated animals, and 16 animals overall. One animal in the non-treated group was euthanized directly following MCAO surgery due to post-operative complications and was removed from the study due to pre-defined exclusion criteria. One animal in the treatment group was euthanized at day 7 post-MCAO due to an intractable leg injury and was excluded from survival and correlation analysis at day 84 post-MCAO according to determined criteria but was included in NSC EV midline shift average calculation. Lastly, one animal in the NSC EV treatment group was retrospectively excluded from all analysis due to discovery of a non-treatment, surgery-related Trueperella abscess determined on post-mortem histopathological examination. This left 5 animals in the NSC EV group and 8 animals in the non-treated group for all correlation and survival analyses. Gait data was collected on 65 parameters (Supplementary Table 1) using GAITFour software version 4.9 × 5 (GaitRite Quadruped Gait Analysis System, NJ) on days 1, 3, 7, 14, 28, 56, and 84 post-MCAO. Open-field behavior data was collected on 25 parameters (Supplementary Table 1) through EthoVision XT Version 11.5 (Noldus Information Technologies, Inc.) on days 1, 3, 7, 21, and 84 [34]. Following exclusion criteria and animal death, there remained 5 animals in the non-treated and 4 animals in the NSC EV–treated group at day 84 post-MCAO. All gait and behavior data was normalized for each animal individually, based on average values recorded during 3 gait and 1 behavior pre-stroke trials.

### Magnetic Resonance Imaging Analysis

MRI was performed on days 1 and 84 post-MCAO on a Siemens 3.0 Tesla Magnetom Avanto MRI system. Utilizing the previously described surgical anesthesia protocol [35], MRI of the cranium was performed using a 12-channel head coil, 25 cm in diameter, with the pig positioned in supine recumbency. Standard multiplanar magnetic resonance (MR) brain imaging sequences were acquired including T2FLAIR, T2W, DWI, and DTI. T2FLAIR, T2W, DWI, and ADC maps were analyzed using Osirix software (version 5.8.5, Pixme, Geneva, Switzerland). DTI and computed FA values were analyzed using ImageJ software. Analysis of MLS was performed by two trained blinded independent raters on coronal and axial planes on T2-weighted MR images at the level of the septum pellucidum. To determine inter-rater reliability, Pearson’s correlation was conducted on MLS measurements in axial and coronal planes by two raters in both NSC EV–treated and non-treated animals. Pearson’s correlation of raters’ measurements showed a NSC EV axial of *R*^2^ = 0.679 (*p* = 0.0228), NSC EV coronal of *R*^2^ = 0.841 (*p* = 0.0036), non-treated axial of *R*^2^ = 0.970 (*p* < 0.0001), and non-treated coronal of *R*^2^ 0.936 (*p* < 0.001) indicating that their measurements were significantly related. Average MLS values of both raters per animal were utilized for all correlation analysis.

#### Midline Shift Analysis

**Fig. 1:**
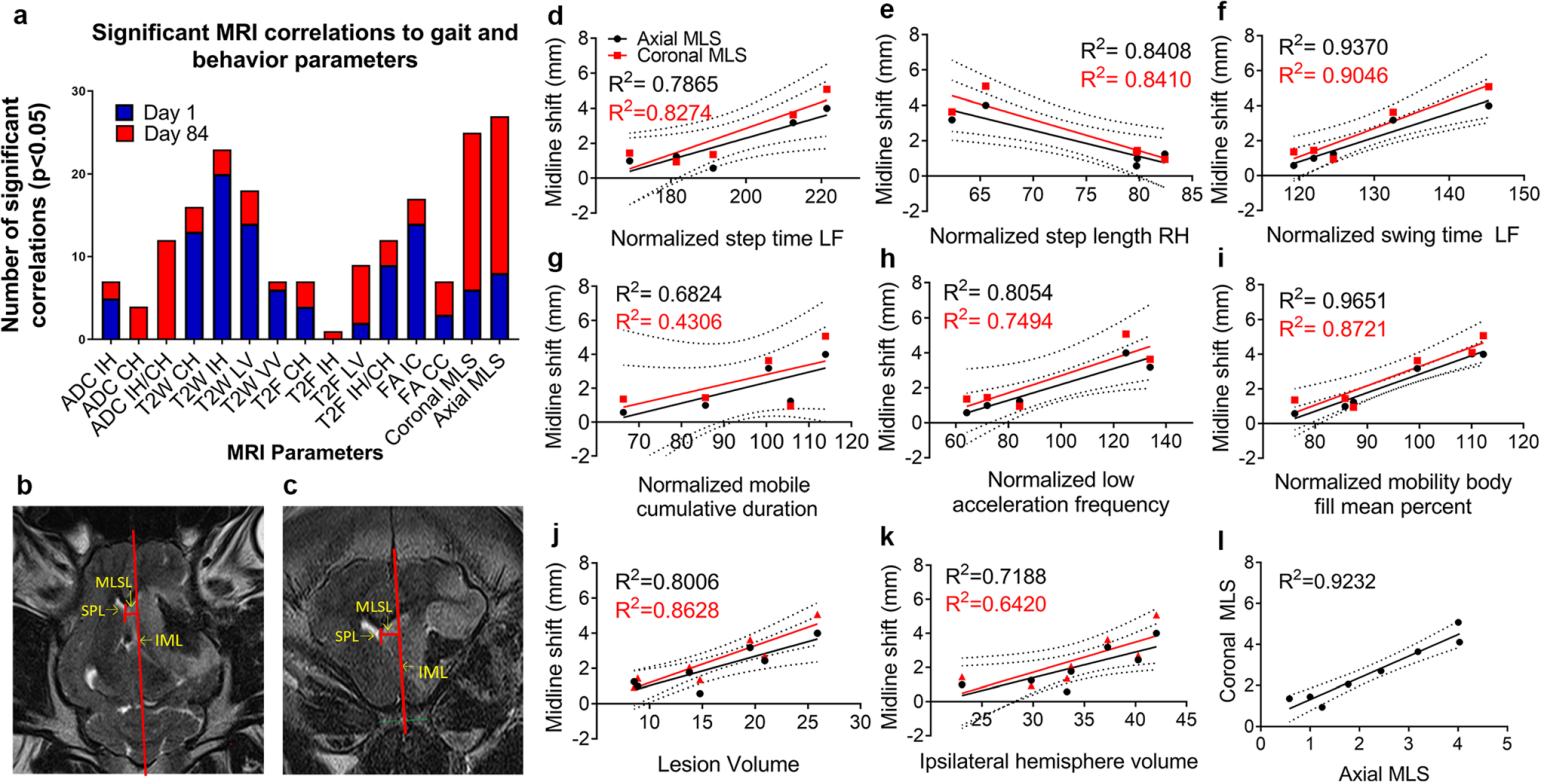
Midline shift is significantly correlated to measurements of functional outcomes. **a** A subset of measured gait and behavior parameters (*n* = 90) significantly correlated with MRI measurements at day 1 (blue) and day 84 (red) post-MCAO (Pearson’s product-moment correlations, *p* < 0.05). Measurements of MLS in the axial (**b**) and coronal (**c**) plane. IML: ideal midline, SPL: septum pellucidum line, MLSL: midline shift line. Linear regression of axial and coronal MLS showed significant correlations with normalized step time in the left front (**d**, cor: *R*^2^ = 0.8274, *p* = 0.0322; ax: *R*^2^ = 0.786519, *p* = 0.0449), normalized step length right hind (**e**, cor: *R*^2^ = 0.840988, *p* = 0.0283; ax: *R*^2^ = 0.840809, *p* = 0.0284), normalized swing time left front (**f**, cor: *R*^2^ = 0.9048, *p* = 0.0129; ax: *R*^2^ = 0.937, *p* = 0.0068), normalized mobile cumulative duration (**g**, cor: *R*^2^ = 0.430566, *p* = 0.1570; ax: *R*^2^ = 0.682416, *p* = 0.0427), normalized low acceleration (**h**, cor: *R*^2^ = 0.74939, *p* = 0.0259; ax: *R*^2^ = 0.805375, *p* = 0.0152), normalized body fill mean percent (**i**, cor: *R*^2^ = 0.872088, *p* = 0.0064; ax: *R*^2^ = 0.965138, *p* = 0.0005), lesion volume (**j**, cor: *R*^2^ = 0.862796, *p* = 0.0009; ax: *R*^2^ = 0.800643, *p* = 0.0027), ipsilateral hemisphere volume (**k**, cor: *R*^2^ = 0.642037, *p* = 0.0168; ax: *R*^2^ = 0.718771, *p* = 0.0078), and axial and coronal MLS (**l**, *R*^2^ = 0.9224, *p* = 0.0001)

Three linear measurements were utilized in the analysis [40, 41]. First, the ideal midline was drawn for reference in the axial (IML; Fig. 1b) and coronal plane (IML; Fig. 1c). Next, a line was drawn down the length of the septum pellucidum (SPL; Fig. 1b, c). Length of the SPL was measured, and the exact middle point was marked. Next, midline shift line (MLSL; Fig. 1b, c) was drawn exactly 90° from the IML to the marked center of the SPL. Finally, the length of MLS line was measured and recorded as the degree of MLS. Extreme studentized deviate test was performed on all MLS values in NSC EV and non-treated animals in both axial and coronal planes for each group, and after confirmation of normal distribution by Shapiro-Wilk goodness-of-fit test, no outliers were found in any group (Supplemental Table 2). Degree of MLS was then used for comparison between NSC EVs and non-treated groups. Within NSC EV–treated and non-treated groups, MLS was either defined as “high,” being above the mean, or “low” being below the mean MLS in a given group and plane. In the non-treated animals, the mean MLS in the axial orientation was 2.283 mm, categorizing 4 animals into the HMLS group and 4 animals into the LMLS group. In NSC EV–treated animals, the mean MLS in the axial orientation was 2.125 mm, categorizing 2 animals into the HMLS group and 3 animals into the LMLS group.

#### Herniation Analysis

All foremen magnum and transtentorial herniation analysis was performed on midsagittal T2W MR images as described [42]. Briefly, bony landmarks were utilized to create three linear measurements: skull length line (SLL; Fig. 4a, b), transtentorial line to the rostral most point along the ventral aspect of the cerebellum (TTX; Fig. 4a, b), and the most caudal point on the ventral aspect of the cerebellum to the foramen magnum line (FMX; Fig. 4a, b). TTX and SLL were utilized to quantify caudal transtentorial herniation (CTH = TTX/SLL; Fig. 4c, d) and FMX and SLL were used to quantify foramen magnum herniation (FMH = FMX/SLL; Fig. 4e, f).

### Modified Rankin Score Post-MCAO

Pigs were assessed by a trained rater and assigned a mRS score pre-MCAO through day 6 post-MCAO [25, 43, 44]. Possible scores ranged from 0 (no residual stroke symptoms) to 6 (death) and were evaluated across the entire score range as a non-continuous variable. This clinical scale was adapted to better capture functional and behavioral recovery specifically in pigs following MCAO and is further described in the Supplemental Materials (Supplementary Table 5).

### Statistical Analysis

**Fig. 2:**
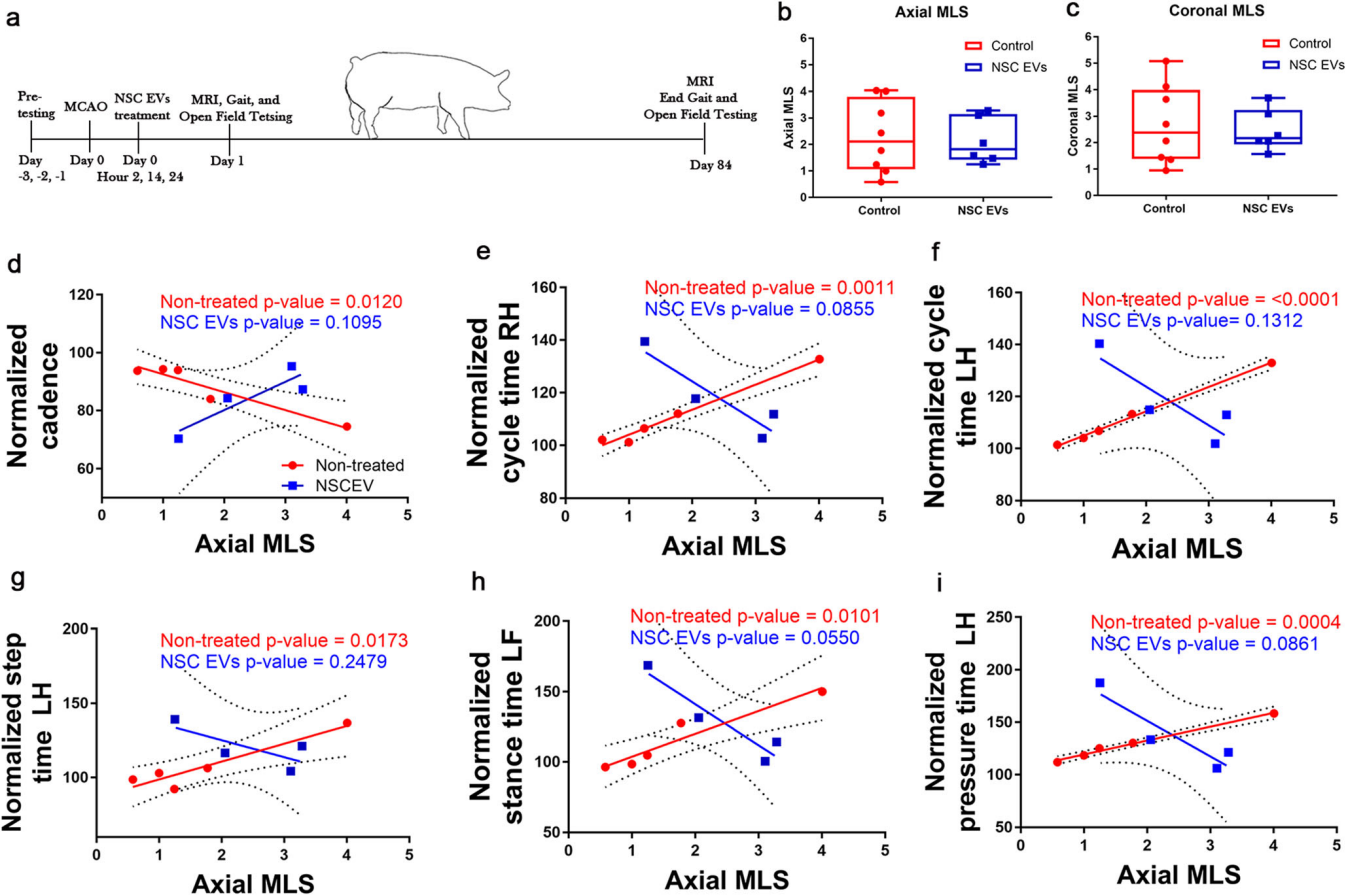
NSC EV treatment disrupts characteristic correlations of midline shift with chronic day 84 functional outcomes measurements. **a** Animals were divided into NSC EV treatment or PBS groups and received treatment or vehicle at 2, 14, and 24 h post-MCAO. MRI was conducted at day 1 and day 84 post-MCAO. Gait and behavior testing was conducted during the intervening 12 weeks as diagramed. Box and whisker plots of average degree of MLS in the axial (**b**) (*p* = 0.8079) and coronal (**c**) (*p* = 0.7587) planes between NSC EV–treated and non-treated groups showed no significant difference. The box represents the interquartile range, the solid horizontal line represents the mean, and whiskers reach to 5th and 95th percentiles. Linear regression of day 1 post-MCAO axial MLS of non-treated animals showed strong correlations at day 84 post-MCAO while NSC EV–treated animals did not in functional measurements of cadence (**d**, non-treated: *R*^2^ = 0.908765, *p* = 0.0120; NSC EVs: *R*^2^ = 0.793059, *p* = 0.1095), cycle time in the right hind limb (**e**, non-treated: *R*^2^ = 0.981288, *p* = 0.0011; NSC EVs: *R*^2^ = 0.836310, *p* = 0.0855), cycle time in the left hind limb (**f**, non-treated: *R*^2^ = 0.996347, *p* < 0.0001; NSC EVs: *R*^2^ = 0.7512, *p* = 0.01312), step time in the left hind limb (**g**, non-treated: *R*^2^ = 0.884398, *p* = 0.0173; NSC EVs: *R*^2^ = 0.563012, *p* = 0.2497), stance time in the left front limb (**h**, non-treated: *R*^2^ = 0.918774, *p* = 0.0101; NSC EVs: *R*^2^ = 0.927527, *p* = 0.1735), and pressure time in the left hind limb (**i**, non-treated: *R*^2^ = 0.991121, *p* = 0.0004; NSC EVs: *R*^2^ = 0.835284, *p* = 0.0861)

**Fig. 3:**
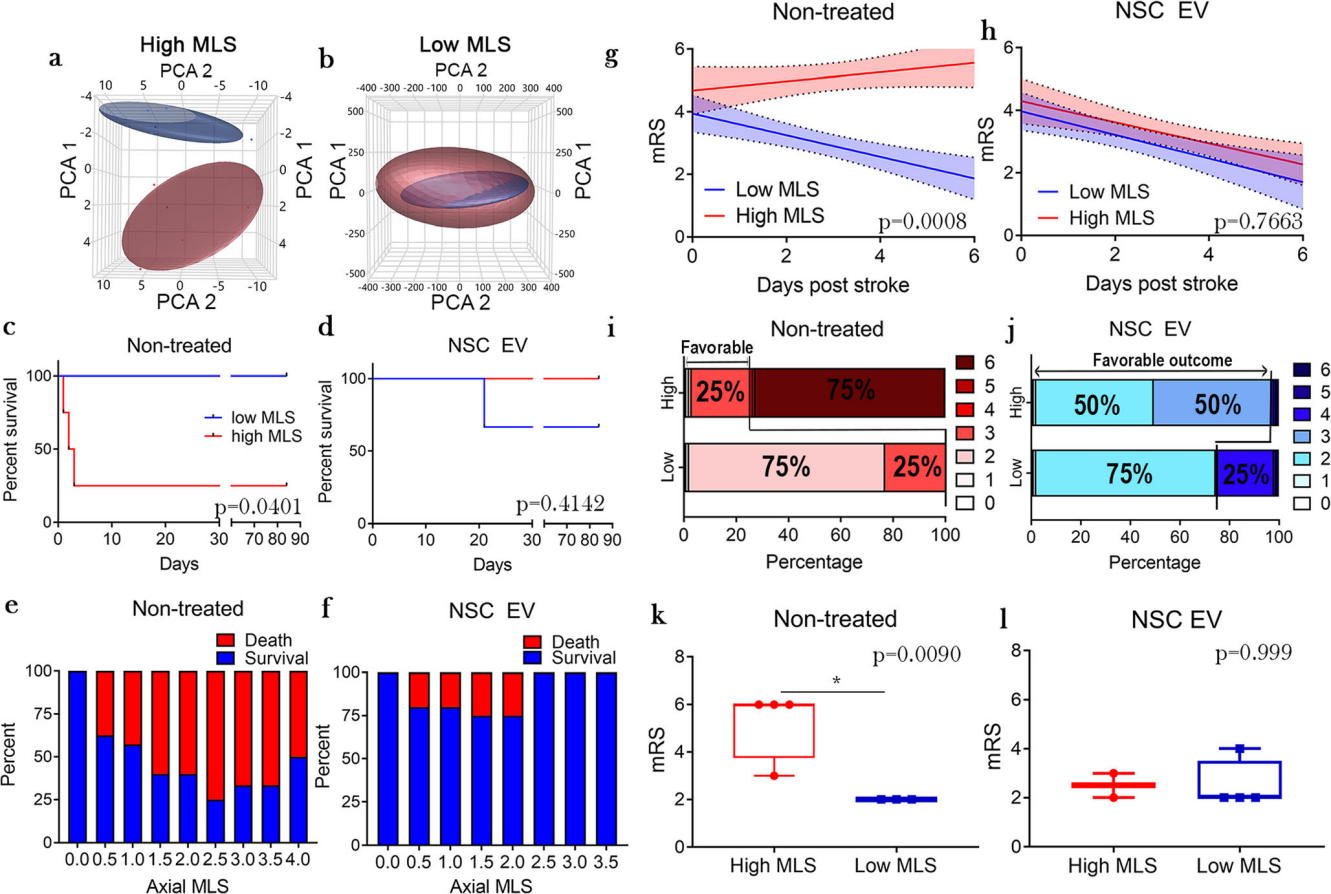
NSC EV treatment disrupts relationship of HMLS with poor survival and mRS score. PCA of all gait parameters measured from day 0 through day 14 post-MCAO in animals with HMLS (**a**) and LMLS (**b**). Non-treated animals with HMLS (**a**, red) are separated from NSC EVs animals HMLS (**a**, blue), while there is no separation of non-treated and NSC EVS animals with LMLS (**b**, red and blue, respectively). Differences in survival between non-treated (**c**, *p* = 0.0401) and NSC EV–treated animals (**d**, *p* = 0.4142) with either HMLS or LMLS as well as differences in survival with increasing MLS in non-treated (**e**) and NSC EV–treated (**f**). MLS versus mRS score days 0–6 post-MCAO in non-treated (**g**) and NSC EV–treated (**h**) animals. In the non-treated group, there was a significant difference in the slopes and speed of recovery of the HMLS (red) and LMLS (blue) groups (*p* = 0.0008), while there was no significant difference in the slopes of the NSC EV HMLS and LMLS lines (*p* = 0.6754). Stacked graphs showing distribution of mRS scores at day 84 post-stroke in non-treated (**i**) and NSC EV–treated (**j**) animals. While non-treated animals had a decreased chance of favorable outcome with HMLS (25%) compared with LMLS (100%), NSC EV–treated animals did not (HMLS = 100%, LMSL = 75%). Box and whisker plots of mRS score at day 6 post-MCAO in non-treated (**k**) and NSC EV (**l**) animals. Non-treated animals with HMLS had a significantly higher mRS score at day 6 post-MCAO (*p* = 0.0090), while NSC EV–treated animals did not (NSC EVS: *p* = 0.9999)

**Fig. 4:**
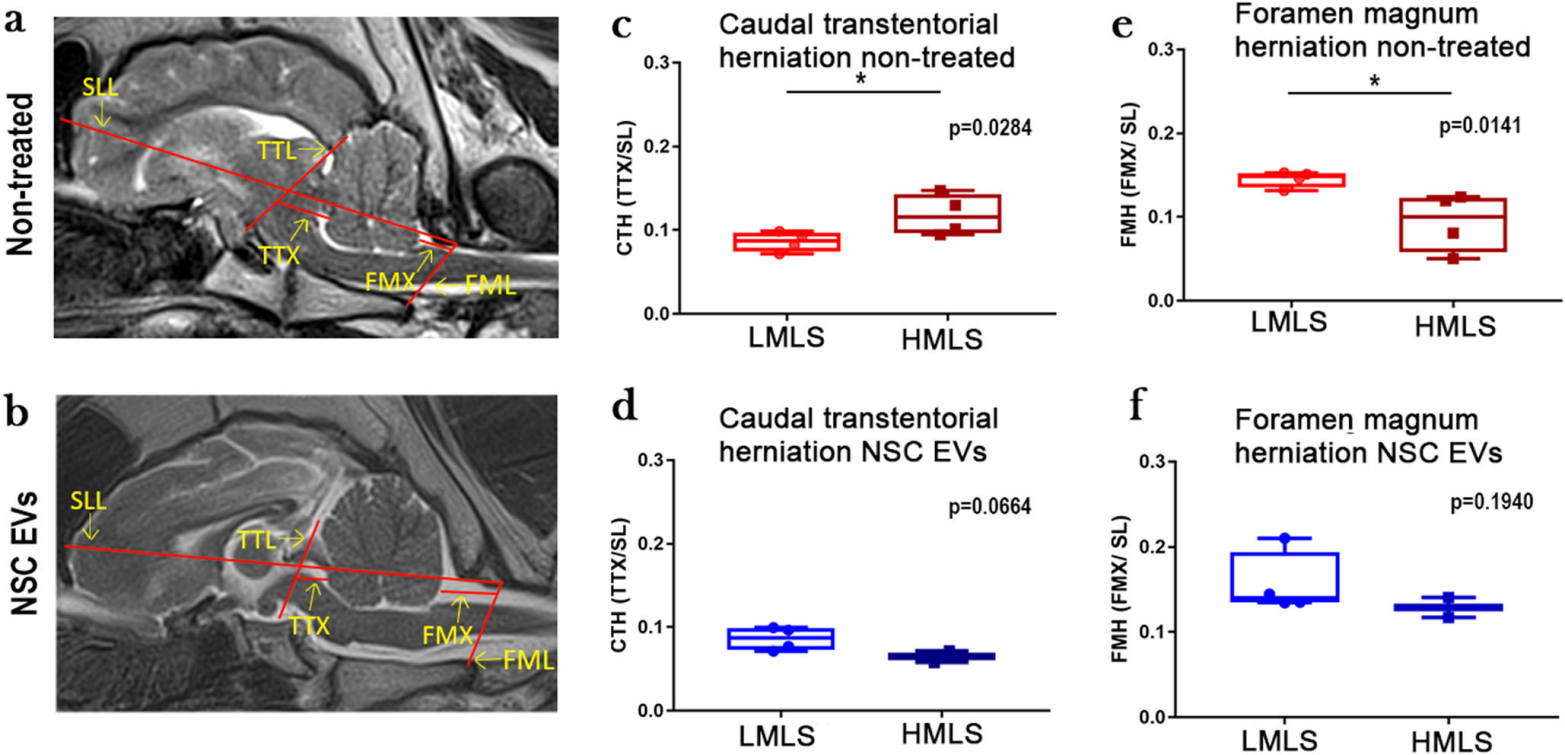
NSC EV treatment inhibits cerebellar herniation in high midline shift animals. Mid-sagittal T2W MR images depicting bony landmarks utilized to discern degree of cerebellar herniation in non-treated (**a**) and NSC EV–treated (**b**) animals. SLL: skull length line, TTL: transtentorial line, FML: foramen magnum line, TTX: transtentorial line to rostral ventral cerebellum, FMX: caudoventral cerebellum to foramen magnum line. There is a significant degree of caudal transtentorial herniation (CTH = TTX/SLL) in between non-treated animals (**c**, *p* = 0.0284) with HMLS and LMLS, while there is not in NSC EV–treated animals (**d**, *p* = 0.0664). Additionally, there is a significant degree of foramen magnum herniation (FMH = FMX/SLL) between HMLS and LMLS non-treated animals (**e**, *p* = 0.0141) while there is not in NSC EV–treated (**f**, *p* = 0.1940)

Pairwise correlations were run between all 15 measured MRI parameters and all 90 gait and behavior parameters (Supplementary Table 1) at day 1 and day 84 post-MCAO using JMP Pro 13.2.0 statistical software (SAS Institute Inc., Carey, NC, USA). Significant pairwise correlations (*p* < 0.05) were recorded for each group at each time point. Total number of significant correlations for each MR imaging measurement at day 1 (acute, blue) and day 84 post-MCAO (chronic, red) were quantified (Fig. 1a). Linear regression was performed in GraphPad Prism 7.04 (GraphPad Software, La Jolla, CA, USA) of coronal and axial MLS vs. normalized gait and behavior parameters as well as mRS scores over days 0–6 post-MCAO in non-treated (*n* = 8) and NSC EV–treated animals (*n* = 6). Coefficient of correlation and *p* value for each significant correlation of axial and coronal MLS at day 1 (gait *n* = 5, behavior *n* = 6; Supplementary Table 3) and at day 84 post-MCAO (non-treated *n* = 5, NSC EVs *n* = 4; Supplementary Table 4) are listed in the Supplemental Materials. Unpaired, two-tailed *t* test was conducted on parametric data including degree of axial and coronal MLS in non-treated (*n* = 8) and NSC EV–treated (*n* = 6) animals (Fig. 2b, c), mRS score at day 6 post-MCAO between HMLS and LMSL in non-treated (*n* = 8) and NSC EV–treated (*n* = 6) animals (Fig. 3k, l). Unpaired, one-tailed *t* test was conducted on degree of caudal transtentorial herniation (Fig. 4c, d) and degree of foramen magnum herniation (Fig. 4e, f) between HMLS and LMLS groups in non-treated (*n* = 8) and NSC EV–treated (*n* = 6) groups. Principal component analysis (PCA) was conducted (Fig. 3a, b) on all 65 gait parameters recorded through day 14 post-MCAO (days 1, 3, 7, 14) with JMP Pro 13.2.0 statistical software (SAS Institute Inc., Carey, NC, USA). Kaplan-Meier curves were generated in GraphPad Prism 7.04 and log-rank Mantel-Cox test was conducted between HMLS and LMLS non-treated (*n* = 8) and NSC EV–treated (*n* = 5) animals.

## Results

### Acute Midline Shift is Highly Correlated with Chronic Functional Outcomes

Multivariate analysis was conducted for all measured MRI parameters at day 1 post-MCAO versus 90 recorded gait and behavior parameters at day 1 and day 84 post-MCAO, in order to determine which MRI parameters were most predictive of functional outcome at acute and chronic time points. T2W ipsilateral hemisphere volume had the highest number of significant (*p* < 0.05) correlations to gait and behavior parameters at day 1 post-MCAO (Fig. 1a). This suggests T2W ipsilateral hemisphere volume may be a predictor of functional deficits in the early acute phase of stroke in a porcine MCAO model. When comparing the same measured MRI parameters to gait and behavior parameters at day 84 post-MCAO, structural measurements of axial and coronal MLS following MCAO had the highest number of significant correlations to recorded gait and behavior parameters with 19 each (Fig. 1a). Axial and coronal MLS had the highest total number of significant (*p* < 0.05) correlations with acute (day 1) and chronic (day 84) measurements (Fig. 1a), suggesting it is the best predictor of overall acute and chronic functional deficits.

Measurements of MLS were conducted in the axial plane (Fig. 1b), more commonly utilized in human clinical literature [40, 41], as well as the coronal plane (Fig. 1c). Coronal plane measurements of MLS are more commonly utilized in rodents [45–47] as measured deviation of the third ventricle. Axial and coronal MLS measured at day 1 post-MCAO were significantly correlated to normalized step time in the left front limb (axial *p* = 0.0449, coronal *p* = 0.0322; Fig. 1d), normalized step length in the right hind limb (axial *p* = 0.0284, coronal *p* = 0.0238; Fig. 1e), and normalized swing time in the left front limb (axial *p* = 0.0068, coronal *p* = 0.0129; Fig. 1f) at day 1 post-MCAO. In these correlations, as MLS increased, so too did step time and swing time in the left front limb, while step length decreased. Additionally, axial and coronal MLS measured at day 1 post-stroke were correlated to normalized acceleration state (axial *p* = 0.0152, coronal *p* = 0.0259; Fig. 1h) and normalized body fill mean percent (axial *p* = 0.0005, coronal *p* = 0.0064; Fig. 1i) measured during open-field behavioral testing at day 1 post-MCAO. These correlations showed an increase in the number of acceleration frequencies in the “low” category as opposed to medium or high accelerations, as well as an increase in body elongation with an increasing MLS. Individually, axial MLS was also correlated to normalized swing percent of cycle in the left hind limb (*p* = 0.0430; Supplementary Table 3), normalized mean pressure of the right front limb (*p* = 0.0432; Supplementary Table 3), and normalized mobile cumulative duration (*p* = 0.0427; Fig. 1g), while coronal MLS was individually correlated to normalized swing time in the right hind limb (*p* = 0.0305; Supplementary Table 3). All significant correlations to gait and behavior measurements at day 1 post-MCAO are listed in Supplementary Table 3. Collectively, gait cycle time and overall activity at low acceleration speeds increased as MLS increased.

In addition to functional measurements post-MCAO, correlations of axial and coronal MLS to other common structural MR measurements were assessed. Axial and coronal measurements of MLS at day 1 post-MCAO were significantly correlated to T2W lesion volume (axial *p* = 0.0128, coronal *p* = 0.0036; Fig. 1j) and ipsilateral hemisphere volume (axial *p* = 0.0369, coronal *p* = 0.0380; Fig. 1k). This is in accordance with the Monro-Kellie doctrine and correlations with previous reports of axial and coronal MLS in other animal models of stroke [38] as well as human stroke patients [48, 49]. Additionally, axial measurements of MLS were correlated to coronal measurements of MLS (*p* < 0.0001; Fig. 1l).

### NSC EV Treatment Alters Normal Correlations between Midline Shift and Chronic Measurements of Functional Outcomes

Stroked animals were randomized into either a non-treated or NSC EV treatment group. Animals assigned to the treatment group received doses of NSC EVs at 2, 14, and 24 h post-MCAO (Fig. 2a) [34]. The average degree of axial and coronal MLS at day 1 post-MCAO was not significantly different between non-treated and NSC EV–treated groups (Fig. 2b, c). However, significant differences were observed between the non-treated and NSC EV–treated group in correlations of axial and coronal MLS to chronic functional outcomes measured at day 84 post-MCAO. Non-treated animals demonstrated a correlation between increases in axial (Fig. 2d–i) and coronal (Supplementary Table 4) MLS and decreases in normalized cadence, or the number of steps over time (non-treated *p* = 0.0120; Fig. 2d), and increases in normalized cycle time in the right hind limb (non-treated *p* = 0.0011; Fig. 2e), normalized cycle time in the left hind limb (*p* < 0.0001; Fig. 2f), normalized step time of the left hind limb (non-treated *p* = 0.0173; Fig. 2g), normalized stance time of the left front limb (non-treated *p* = 0.0101; Fig. 2h), and normalized pressure time in the left hind (non-treated *p* = 0.0004; Fig. 2j). Additional gait and behavior parameters that were significantly correlated to axial and coronal MLS in non-treated animals are listed in Supplementary Table 4. Interestingly, normalized cadence (NSC EVs *p* = 0.1095; Fig. 2d), normalized cycle time in the right hind (NSC EVs *p* = 0.0855; Fig. 2d), normalized cycle time in the left hind limb (NSC EVs *p* = 0.1312; Fig. 2f), normalized step time of the left hind limb (NSC EVs *p* = 0.2497; Fig. 2g), normalized stance time in the left front limb (NSC EVs *p* = 0.0550; Fig. 2h), and normalized pressure time in the left hind limb (NSC EVs *p* = 0.0861; Fig. 2i) were not significantly correlated to MLS in the NSC EV–treated animals. Instead, these parameters tended to show an inverse correlation to that of non-treated animals, indicating a disruption of these established correlations with treatment. Overall, these correlations reveal that an increase in MLS measurements taken 24 h after stroke in either plane is significantly correlated with functional impairments in animals that received no treatment(s); however, NSC EV treatment disrupted the correlation of MLS such that larger MLS in NSC EV–treated animals did not lead to the same functional deficits.

### NSC EV Treatment Provides Significant Survival and Functional Benefit to Animals with High Midline Shift

Following observed differences in gait correlations of non-treated and NSC EV–treated animals, especially those with high MLS, animals were binned into either high or low MLS (HMLS and LMLS, respectively) groups, similar to what is done clinically when defining malignant edema in human patients [50]. Due to differences in neuroanatomical size between pigs and humans, the exact clinical criteria for MLS definitions could not be implemented and were therefore adapted to better suit pigs. For the purposes of this study, the distinction of HMLS and LMLS was assigned according to position relative to the overall group mean (average MLS in axial non-treated = 2.283 mm, coronal non-treated = 2.670 mm, axial NSCEV = 2.125 mm, coronal NSCEV = 2.459 mm; Fig. 2b, c). Once separated to LMLS and HMLS groups, PCA analysis of all gait parameters up to day 14 post-MCAO revealed a separation between non-treated and NSC EV–treated animals with HMLS (Fig. 3a). In non-treated animals, there was a significant increase (*p* = 0.0401 log-rank Mantel-Cox test) in survival of animals with a LMLS relative to animals with a HMLS (Fig. 3c). There was no significant difference (log-rank Mantel-Cox test *p* = 0.4142) between survival of HMLS and LMLS animals in the NSC EV–treated group (Fig. 3d). One animal in the NSC EV LMLS group expired 21 days post-MCAO due to meningoencephalitis, causing a lower but non-significant survival percentage in the NSC EV–treated LMLS group compared with the HMLS group. When comparing survival with incrementally increasing MLS, non-treated animals with an axial MLS greater than 3.5 mms only had a 33% survival rate (Fig. 3e), while NSC EV–treated animals had a 100% survival rate (Fig. 3f). These results suggest that although NSC EV treatment does not significantly alter the degree of MLS 24 h post-MCAO, it does increase the chance of survival following a large MLS alteration, which is usually detrimental to survival.

Changes in motor function and behavior over the first 6 days post-MCAO were assessed utilizing a pig-specific adaptation of the modified Rankin scale (mRS; adaptations listed in Supplementary Table 5). In non-treated animals, those with LMLS had a significant decrease in mRS score over days 0–6 (*m* = − 0.3465 ± 0.08361, non-zero slope *p* value = 0.0005; Fig. 3g), while those with HMLS did not (*m* = 0.1473 ± 0.1033, non-zero *p* value = 0.1665; Fig. 3g). When compared directly, LMLS non-treated animals had a statistically faster recovery measured by mRS relative to HMLS animals (*p* = 0.0008; Fig. 3g). However, there was no significant difference in speed of recovery of LMLS (*m* = − 0.2815 ± 0.08607, non-zero *p* = 0.004; Fig. 3h) and HMLS (*m* = − 0.3378 ± 0.08806, non-zero *p* = 0.0028; Fig. 3h) in NSC EV–treated animals (*p* = 0.6754; Fig. 3h).

When comparing distribution of mRS scores of non-treated animals at day 6 post-MCAO, 75% of animals with HMLS were defined as having poor clinical outcome scores (mRS ≥ 4; Fig. 3i). In comparison, 0% of NSC EV–treated animals with HMLS were defined as having poor clinical outcome scores (Fig. 3j). Lastly, when comparing average mRS scores at day 6 post-MCAO, there was a significant difference in mRS scores of non-treated animals with HMLS and LMLS (non-treated *p* = 0.0090; Fig. 3k), while there was no significant difference in mRS scores of NSC EV treatment animals with HMLS and LMSL (NSC EVS *p* = 0.9999; Fig. 3l). Taken together, these results suggest HMLS significantly correlates with decreased recovery speed as measured by mRS in non-treated animals, while NSC EV treatment eliminates the difference in speed of recovery between LMLS and HMLS animals.

### NSC EV Treatment Inhibits Cerebellar Herniation with Increasing Midline Shift

The degree of cerebellar herniation is often a vital clinical measurement, as the cerebellum contains centers involved in pneumotaxis and cardiovascular regulation which are often impinged upon with herniation [51, 52]. Cerebellar herniation was measured on sagittal T2W image slices as previously described in canine and feline models (Fig. 4a, b) [42]. Non-treated animals with HMLS had a significant increase in caudal transtentorial herniation (*p* = 0.0284; Fig. 4c) and a significant decrease in foramen magnum herniation (*p* = 0.0141; Fig. 4e) compared with non-treated animals with LMLS, indicating greater herniation of the cerebellum away from midbrain structures and towards the foramen magnum. In NSC EV–treated animals, there was no significant difference between animals with HMLS and LMLS in the degree of caudal transtentorial (*p* = 0.0664; Fig. 4d) or foramen magnum herniation (*p* = 0.1940; Fig. 4f). This indicates that NSC EV treatment effectively protects against cerebellar herniation in animals with HMLS.

## Discussion

There are multiple barriers to clinical translation of effective ischemic stroke therapeutics; one of which is the identification of an MRI-detectable structural parameter that can non-invasively predict functional outcome [14, 53–55]. Once identified and validated, these predictive biomarkers could then be utilized to assess clinical efficacy of novel therapeutics through divergence from established functional correlations [52]. Out of multiple MRI parameters assessed through an unbiased multiparametric approach, MLS at day 1 post-MCAO, measured in the axial or coronal plane, proved to be the most predictive of gait and behavioral outcomes at days 1 and 84 post-ischemic stroke. This study supports utilization of MLS as a reliable, efficient, translational, and predictive indicator of functional recovery if no interventional therapies are provided in a porcine stroke model. Furthermore, MLS was a useful distinguishing parameter to identify the efficacy of NSC EV treatment in a subpopulation of animals, those with HMLS, which had improved prognostic outcomes over correlative projections. This is the first case demonstrating the clinical relevance of MLS as a predictor of measurable functional outcomes in an animal model.

Although modifications to the gait cycle, such as decreased cadence, are observed in animal models [56] and patients after stroke [57, 58], this study uniquely established that the degree of altered gait increased tandemly with the level of MLS. Open-field behavior testing of pigs also revealed trends in parameters measured at day 1 post-MCAO with acute MLS. Measures of hyperactivity, elongation, and mobility significantly increased with increasing MLS. While seemingly counterintuitive animal studies have documented increases in motor activity acutely following stroke [59], mostly attributed to increased circling behavior which is commonly observed in animal models [60] and was observed in our porcine model (Supplementary Fig. 1). MLS prospectively predicted measurable and graded changes in gait and behavior and might be used in the future to personalize a patients’ prescribed course of rehabilitation after stroke.

The utility of the MLS parameter was further evidenced with incorporation of functional outcome data following NSC EV therapeutic intervention. As expected, the non-treated animals in the HMLS group exhibited reduced survival rates compared with animals with LMLS. However, NSC EV treatment of animals following stroke resulted in a statistically significant, clinically relevant deviation from traditional correlations that associate increasing MLS with increasing functional impairments and mortality. These clinically relevant results demonstrate that NSC EV treatment is able to protect against characteristic functional impairments following stroke even in instances of large parenchyma distortions, suggesting enhanced therapeutic effect for patients with large disruptions and decreased need for more invasive interventions. Furthermore, given the high mortality rates associated with ischemic stroke patients, NSC EV treatment may promote overall recovery and improve survival, even in patients with high MLS and typically poor prognosis.

While MLS has been utilized clinically, it traditionally is regarded as a measure of edema and swelling following stroke incident [61], not as a predictive tool for motor function prognosis or rehabilitation planning. Instead, it is often utilized to identify malignant transformation of middle cerebral artery infarcts [62, 63]. Once determined, this transformation is prescriptive of severe and invasive interventions such as hemicraniectomy, and is often associated with a staggering increase in mortality [64]. Here, NSC EV–treated animals with HMLS, at ranges comparable to those necessitating surgical interventions in humans, had indistinguishable mRS scores and survival rates from animals with LMLS. Therefore, NSC EV treatment may prove to be a less-invasive alternative for patients with massive infarcts compared with current standards of care such as surgical decompression or barbiturate coma therapy [65]. Less-invasive alternative approaches such as NSC EV treatment would also be highly desirable in clinical cases involving elderly comorbid patients [66].

While trends and correlations between MLS and mRS scores [41, 52, 64, 67] and survival [68, 69] at acute and chronic time points have been documented in clinical studies, this is the first time these correlations were identified in an animal model. Additionally, this was the first time these correlations were disrupted with a therapeutic treatment in a preclinical study, showing improved prognosis with treatment. Furthermore, this unique large animal model of stroke allowed for a more traditional measurement of MLS over other animal models. While human MLS measurements can be made at various levels and orientations, clinicians have favored measuring the shift of the septum pellucidum at the level of the fornix in the axial plane [68, 70, 71]. These same measurements, however, cannot be replicated in rodents, and are constrained to deviation of rodents’ third ventricle in the coronal plane [38, 45, 72]. While clinically traditional measurements of MLS have been accomplished in larger animal models of stroke, such as ovine, attempts to correlate these measurements to specific functional outcomes have not been reported in animal models [73]. Given the significance of MLS determinations in a human clinical setting, our porcine model reinforces the importance and now the predictive potential of measuring a shifted septum pellucidum at the level of the fornix in the axial plane.

Lastly, the presence of foramen magnum and caudal transtentorial herniation was shown to correspond with HMLS in non-treated animals, while NSC EV–treated animals did not exhibit a difference in the degree of herniation between those with LMLS and HMLS. This distinction between non-treated and NSC EV–treated animals suggests that NSC EVs may reduce swelling following large stroke insults, leading to less herniation [34]. Preservation of these neuroanatomical relationships, particularly regarding the cerebellum and brainstem, which is known to house centers necessary for thermoregulation [74], cardiovascular regulation [75], and respiration [76], could attribute to the survival and functional benefits observed in NSC EV–treated animals. In a previous rodent embolic model of stroke, IN-111–labeled NSC EVs were found to be present in the infarct region 1 h after EV administration and cleared from the infarct area by 24 h post-administration [33]. This demonstrates that EVs are able to cross the damaged BBB to the area of infarct for possible local effects. NSC EVs were also found systemically in the lung’s liver and spleen, opening the possibility for a systemic-based mechanism of action. Therefore, NSC EVs may be acting through local and/or systemic mechanisms to decrease edema and herniation following stroke in animals with HMLS. These results must be interpreted while considering the limitations of this study with one being the small sample number of animals in LMLS and HMLS groups. Future studies should include larger cohorts of animals to further expand upon these findings. Together, these herniation results with survival and mRS results suggest that NSC EVs serve as a less-invasive alternative therapeutic in cases of high MLS and therefore intracranial pressure (ICP).

## Conclusion/ Summary

The correlations of MLS to functional outcomes served as valuable parameters for distinguishing stroke severity and therapeutic efficacy in this study. These results expand on the current clinical utilization of MLS as a corollary to survival and mRS score through identification of significant correlations to limb-specific gait alterations after stroke. Patient MLS could prove to be a non-invasive and useful tool to develop targeted rehabilitation regimens through specific prognostic correlations, as well as a key measurement to identify disruptive and effective intervention therapeutics, such as NSC EV treatment.

## Electronic Supplementary Material

ESM 1 (DOCX 523 kb)

## Data Availability

The datasets generated and/or analyzed during the current study are available from the corresponding author on reasonable request.
