# Supplementary material for: Neural Stem Cell Extracellular Vesicles Disrupt Midline Shift Predictive Outcomes in Porcine Ischemic Stroke Model
Source: Transl Stroke Res. 2019 Dec 6;11(4):776–88. doi: 10.1007/s12975-019-00753-4 (PMC7340639; doi:10.1007/s12975-019-00753-4)
Supplement: Supplementary file 1 — (DOCX 523 kb) [file 12975_2019_753_MOESM1_ESM.docx]

**Supplemental Data
Supplementary Table 1 –All specific gait, behavior, and MRI parameters measured and utilized for correlation analysis.**

| **Measured Parameters** | | | |
| --- | --- | --- | --- |
| **Gait** | | **Behavior** | **MRI** |
| Normalized Velocity | Normalized Number of Sensors Left Front | Clockwise Circles | ADC Ipsilateral Hemisphere (IH) |
| Normalized Cadence | Normalized Number of Sensors Right Front | Counterclockwise Circles | ADC Contralateral Hemisphere (CH) |
| Normalized Step Time Left Front | Normalized Number of Sensors Left Hind | Normalized Distance moved center point (m) | ADC Ipsilateral Hemisphere (IH)/Contralateral Hemisphere (CH) |
| Normalized Step Time Right Front | Normalized Number of Sensors Right Hind | Normalized Velocity (m/s) | T2W Contralateral Hemisphere (CH) Volume |
| Normalized Step Time Left Hind | Normalized Total Scaled Pressure Left Front | Normalized Movement- moving cumulative duration (s) | T2W Ipsilateral Hemisphere (IH) Volume |
| Normalized Step Time Right Hind | Normalized Total Scaled Pressure Right Front | Normalized Movement- not moving cumulative duration (s) | T2W Lesion Volume |
| Normalized Step Length (cm) Left Front | Normalized Total Scaled Pressure Left Hind | Normalized Mobility state - highly mobile frequency | T2W Ventricle Volume |
| Normalized Step Length (cm) Right Front | Normalized Total Scaled Pressure Right Hind | Normalized Mobility state - highly mobile cumulative duration (s) | T2F Contralateral Hemisphere (CH) Volume |
| Normalized Step Length (cm) Left Hind | Normalized Mean Pressure Right Front | Normalized Mobility state - mobile Standard E(s) | T2F Ipsilateral Hemisphere (IH) volume |
| Normalized Step Length (cm) Right Hind | Normalized Mean Pressure Left Front | Normalized Mobility state- mobile frequency | T2F Lesion volume |
| Normalized Cycle Time Left Front | Normalized Mean Pressure Left Hind | Normalized Mobility state- mobile cumulative duration (s) | T2F Ipsilateral/ Contralateral x100 |
| Normalized Cycle Time Right Hind | Normalized Mean Pressure Right Hind | Normalized Mobility state- Immobile Standard E (s) | FA Internal Capsule |
| Normalized Cycle Time LH | Normalized Pressure time Left Hind | Normalized Mobility state- immobile frequency | FA Corpus Callosum |
| Normalized Cycle Time Right Hind | Normalized Pressure time Right Front | Normalized Mobility state - immobile cumulative duration (s) | Coronal MLS |
| Normalized Stride Length Left Front | Normalized Pressure time Left Hind | Normalized Acceleration - minimum (m/s^2) | Axial MLS |
| Normalized Stride Length Right Front | Normalized Pressure time Right Hind | Normalized Acceleration- maximum (m/s^2) |  |
| Normalized Stride Length Left Hind |  | Normalized Acceleration state- high acceleration frequency |  |
| Normalized Stride Length Right Hind | Normalized Gait Lameness Score Left Front | Normalized Acceleration state- high acceleration – cumulative duration (s) |  |
| Normalized Swing Percent of Cycle Left Front | Normalized Gait Lameness Score Right Front | Normalized Acceleration state- low acceleration -frequency |  |
| Normalized S Swing Percent of Cycle Right Front | Normalized Gait Lameness Score Left Hind | Normalized Acceleration state - low acceleration cumulative duration (s) |  |
| Normalized Swing Percent of Cycle Left Hind | Normalized Gait Lameness Score Right Hind | Normalized Body elongation- body fill mean (%) |  |
| Normalized Swing Percent of Cycle Right Hind | Normalized Hind Reach Left | Normalized Body elongation state- stretched cumulative duration (s) |  |
| Normalized Swing Time (sec) Left Front | Normalized Hind Reach Right | Normalized Body elongation state- normal cumulative duration (s) |  |
| Normalized Swing Time (sec) Right Front | Normalized Total Pressure Index Left Front | Normalized Body elongation state- contracted cumulative duration (s) |  |
| Normalized Swing Time (sec) Left Hind | Normalized Total Pressure Index Right Front | Normalized Mobility - body fill mean (%) |  |
| Normalized Swing Time (sec) Right Hind | Normalized Total Pressure Index Left Hind |  |  |
| Normalized Stance Percent of Cycle Left Front | Normalized Total Pressure Index Right Hind |  |  |
| Normalized Stance Percent of Cycle RF | Normalized Step/Stride Left Front |  |  |
| Normalized Stance Percent of Cycle LH | Normalized Step/Stride Right Front |  |  |
| Normalized Stance Percent of Right Hind | Normalized Step/stride Left Hind |  |  |
| Normalized Stance Time (sec) Left Front | Normalized Step/Stride Right Hind |  |  |
| Normalized Stance time (sec) Right Front | Norm Stance Time (sec) Right Hind |  |  |
| Normalized Stance Time (sec) Left Hind |  |  |  |

**Supplemental Table 2 – Table of MLS measurements, average, and outlier test results for each treatment group and orientation**

| Treatment group | Orientation | Mean (mm) | Shapiro-Wilk Goodness-of-fit test for normality | MLS value (mm) | Critical value of z | Significant outlier |
| --- | --- | --- | --- | --- | --- | --- |
| NSCEV | Coronal | 2.4592 | P=0.4692, null accepted and normally distributed | 3.0945 | 0.809325 | no |
|  |  |  |  | 1.5655 | 1.138841 | no |
|  |  |  |  | 2.0655 | 0.501476 | no |
|  |  |  |  | 3.6960 | 1.575551 |  |
|  |  |  |  | 2.0590 | 0.509756 | no |
|  |  |  |  | 2.2745 | 0.235239 | no |
|  | Axial | 2.1251 | P=0.1937, null accepted and normally distributed | 3.1030 | 1.123736 | no |
|  |  |  |  | 1.2505 | 1.004994 | no |
|  |  |  |  | 1.4780 | 0.743571 | no |
|  |  |  |  | 3.2850 | 1.332874 | no |
|  |  |  |  | 1.5810 | 0.625213 | no |
|  |  |  |  | 2.0530 | 0.82832 | no |
| Non-treated | Coronal | 2.6695 | P=0.5632, null accepted and normally distributed | 2.0660 | 0.407595 | no |
|  |  |  |  | 2.7030 | 0.022625 | no |
|  |  |  |  | 4.1120 | 0.974242 | no |
|  |  |  |  | 5.0760 | 1.625313 | no |
|  |  |  |  | 1.4495 | 0.823969 | np |
|  |  |  |  | 3.6405 | 0.655798 | no |
|  |  |  |  | 1.3630 | 0.882390 | no |
|  |  |  |  | 0.9460 | 1.164025 | no |
|  | Axial | 2.2826 | P=0.3933, null accepted and normally distributed | 1.7740 | 0.376744 | no |
|  |  |  |  | 2.4385 | 0.115458 | no |
|  |  |  |  | 4.0315 | 1.295412 | no |
|  |  |  |  | 4.0065 | 1.276894 | no |
|  |  |  |  | 0.9995 | 0.950425 | no |
|  |  |  |  | 3.1880 | 0.670622 | no |
|  |  |  |  | 0.5805 | 1.260784 | no |
|  |  |  |  | 1.2425 | 0.770433 | no |

**Supplementary Table 3 – Correlation of 24-hour coronal and axial midline shift at day 1 post-MCAO**

|  | Non-treated (n=5) | | | | NSC EV (n=3) | | | |
| --- | --- | --- | --- | --- | --- | --- | --- | --- |
|  | Coronal | | Axial | | Coronal | | Axial | |
| Parameter | Coefficient of correlation (r) | p-value | Coefficient of correlation (r) | p-value | Coefficient of correlation (r) | p-value | Coefficient of correlation (r) | p-value |
| Normalized Step Time Left Front | **0.9096** | **0.0322** | **0.8869** | **0.0449** | 0.765 | 0.4455 | 0.8527 | 0.3499 |
| Norm Step Length (cm) Left Front | 0.8408 | 0.0744 | -0.7767 | 0.1223 | -0.9407 | 0.2203 | -0.9809 | 0.1247 |
| **Norm Step Length (cm) Right Hind** | **0.9171** | **0.0283** | **-0.917** | **0.0284** | -0.9289 | 0.2416 | 0.8187 | 0.3894 |
| Norm Swing % of Cycle Left Hind | 0.8307 | 0.0815 | **-0.8902** | **0.043** | -0.753 | 0.4572 | -0.843 | 0.3616 |
| Norm Swing Time (sec) Left Front | **0.9511** | **0.0129** | ***0.968*** | ***0.0068*** | 0.688 | 0.5169 | 0.7888 | 0.4214 |
| Norm Swing Time (sec) Right Hind | **0.9128** | **0.0305** | 0.8269 | 0.0842 | 0.9135 | 0.2668 | 0.964 | 0.1712 |
| Norm mean Pressure Right Front | 0.7944 | 0.1084 | **-0.8899** | **0.0432** | -0.3595 | 0.7659 | 0.8111 | 0.3977 |
| Norm Hind Reach Left | 0.7413 | 0.1517 | 0.5446 | 0.3425 | 0.9757 | 0.1406 | 0.932 | 0.2362 |
| Clockwise Circles | 0.6809 | 0.2057 | 0.615 | 0.2696 | **0.9987** | **0.0319** | 0.995 | 0.0637 |
| Counterclockwise Circles | 0.3028 | 0.6204 | -0.3428 | 0.5722 | **-0.*9999*** | ***0.007*** | -0.9903 | 0.0886 |
| Normalized Mobility State- mobile cumulative duration (s) | 0.6562 | 0.157 | **0.8261** | **0.0427** | 0.1655 | 0.8941 | 0.3112 | 0.7986 |
| Normalized Acceleration State- low acceleration -frequency (%) | **0.8657** | **0.0259** | **0.8974** | **0.0152** | 0.7895 | 0.4207 | 0.8724 | 0.3251 |
| Normalized Acceleration State- low acceleration -cumulative duration (s) | 0.5031 | 0.309 | -0.3537 | 0.4915 | -0.9807 | 0.1254 | **-0.9989** | **0.0298** |
| Normalized Mobility - body fill mean (%) | ***0.9339*** | ***0.0064*** | ***0.9824*** | ***0.0005*** | 0.1163 | 0.9258 | 0.2636 | 0.8302 |

**Supplementary Table 4 – Correlations of 24-hour coronal and axial midline shift at Day 84 post-MCAO**

|  | Non-treated (n=5) | | | | NSC EV (n=4) | | | |
| --- | --- | --- | --- | --- | --- | --- | --- | --- |
|  | Coronal | | Axial | | Coronal | | Axial | |
| Parameter | Coefficient of correlation (r) | p-value | Coefficient of correlation (r) | p-value | Coefficient of correlation (r) | p-value | Coefficient of correlation (r) | p-value |
| Velocity | -0.6823 | 0.2044 | -0.6298 | 0.2548 | 0.8966 | 0.1034 | **0.9639** | **0.0361** |
| Normalized Cadence | **-0.9467** | **0.0146** | **-0.9533** | **0.012** | 0.797 | 0.203 | 0.8905 | 0.1095 |
| Normalized Step Time Right Front | 0.8397 | 0.0752 | **0.9537** | **0.0119** | -0.7978 | 0.2022 | -0.8892 | 0.1108 |
| Normalized Step Time Left Hind | ***0.9933*** | ***0.0007*** | **0.9404** | **0.0173** | -0.6271 | 0.3729 | -0.7503 | 0.2497 |
| Normalized Step Time Right Hind | 0.6628 | 0.2228 | 0.8206 | 0.0887 | -0.8963 | 0.1037 | **-0.9544** | **0.0456** |
| Normalized Step Length (cm) Left Front | -0.483 | 0.4099 | -0.5363 | 0.3514 | **0.9525** | **0.0475** | ***0.9926*** | ***0.0074*** |
| Normalized Step Length (cm) Left Hind | -0.4423 | 0.4558 | -0.5499 | 0.337 | **0.9851** | **0.0149** | **0.9833** | **0.0167** |
| Normalized Cycle Time Left Front | **0.9454** | **0.0152** | ***0.99*** | ***0.0012*** | -0.837 | 0.163 | -0.9123 | 0.0877 |
| Normalized Cycle Time Right Front | ***0.9717*** | ***0.0057*** | ***0.9866*** | ***0.0019*** | -0.8094 | 0.1906 | -0.8937 | 0.1063 |
| Normalized Cycle Time Left Hind | ***0.963*** | ***0.0085*** | ***0.9982*** | ***<.0001*** | -0.7795 | 0.2205 | -0.8688 | 0.1312 |
| Normalized Cycle Time Right Hind | ***0.9607*** | ***0.0093*** | ***0.9906*** | ***0.0011*** | -0.8338 | 0.1662 | -0.9145 | 0.0855 |
| Normalized Stride Length Left Front | **-0.9353** | **0.0196** | -0.8186 | 0.0902 | 0.9353 | 0.0647 | 0.918 | 0.082 |
| Normalized Stride Length Right Front | -0.5977 | 0.2871 | -0.5797 | 0.3056 | **0.9672** | **0.0328** | **0.9632** | **0.0368** |
| Normalized Swing Percent of Cycle Right Front | -0.655 | 0.2303 | -0.7258 | 0.1651 | 0.9222 | 0.0778 | **0.9755** | **0.0245** |
| Normalized Swing Percent of Cycle Left Hind | **-0.9458** | **0.015** | ***-0.9877*** | ***0.0016*** | ***0.9969*** | ***0.0031*** | **0.975** | **0.025** |
| Normalized Swing Percent of Cycle Right Hind | **-0.8884** | **0.044** | **-0.907** | **0.0336** | **0.9579** | **0.0421** | **0.9866** | **0.0134** |
| Normalized Stance Percent of Cycle Right Front | 0.6237 | 0.2609 | 0.6858 | 0.2012 | -0.9247 | 0.0753 | **-0.9798** | **0.0202** |
| Normalized Stance Percent of Cycle Left Hind | **0.9573** | **0.0105** | ***0.9908*** | ***0.0011*** | ***-0.9965*** | ***0.0035*** | **-0.9802** | **0.0198** |
| Normalized Stance Percent of Cycle Right Hind | **0.9104** | **0.0318** | **0.9043** | **0.035** | -0.9454 | 0.0546 | **-0.9833** | **0.0167** |
| Normalized Stance Time (sec) Left Hind | **0.9134** | **0.0302** | **0.9585** | **0.0101** | -0.8738 | 0.1262 | -0.945 | 0.055 |
| Normalized Stance Time (sec) Right Front | **0.8999** | **0.0375** | **0.9394** | **0.0177** | -0.8777 | 0.1223 | -0.9444 | 0.0556 |
| Normalized Stance Time (sec) Left Hind | ***0.966*** | ***0.0075*** | ***0.9988*** | ***<.0001*** | -0.9364 | 0.0636 | ***-0.9696*** | ***0.0304*** |
| Normalized Stance Time (sec) Right Hind | ***0.9798*** | ***0.0034*** | ***0.9937*** | ***0.0006*** | -0.9049 | 0.0951 | **-0.9579** | **0.0421** |
| Normalized Total Scaled Pressure Left Front | -0.4154 | 0.4867 | -0.4547 | 0.4417 | **0.9663** | **0.0337** | 0.907 | 0.093 |
| Normalized Mean Pressure Right Front | -0.7522 | 0.1425 | -0.7695 | 0.1281 | 0.9395 | 0.0605 | **0.9817** | **0.0183** |
| Normalized Pressure Time Left Front | **0.9535** | **0.0119** | **0.9451** | **0.0153** | -0.6756 | 0.3244 | -0.7973 | 0.2027 |
| Normalized Pressure Time Right Front | ***0.995*** | ***0.0004*** | ***0.9847*** | ***0.0023*** | -0.9162 | 0.0838 | **-0.9747** | **0.0253** |
| Normalized Pressure Time Left Hind | **0.9333** | **0.0205** | ***0.9956*** | ***0.0004*** | -0.8437 | 0.1563 | -0.9139 | 0.0861 |
| Normalized Pressure Time Right Hind | ***0.9864*** | ***0.0019*** | ***0.9787*** | ***0.0037*** | -0.9157 | 0.0843 | **-0.9735** | **0.0265** |
| Normalized Gait Lameness Score Right Hind | -0.516 | 0.3734 | -0.4515 | 0.4453 | **-0.9604** | **0.0396** | -0.8931 | 0.1069 |
| Normalized Total Pressure Index Right Hind | -0.5286 | 0.3597 | -0.469 | 0.4255 | **-0.9673** | **0.0327** | -0.9054 | 0.0946 |
| Normalized Body Elongation State- stretched cumulative duration (s) | -0.4521 | 0.4446 | -0.5931 | 0.2918 | ***0.9919*** | ***0.0081*** | ***0.9966*** | ***0.0034*** |
| Normalized Body Elongation state- normal cumulative duration (s) | 0.3353 | 0.5812 | 0.4947 | 0.3969 | -0.9001 | 0.0999 | **-0.9637** | **0.0363** |

**Supplementary Table 5**- Clinical modified Rankin Scale (mRS) descriptions for each score with pig adaptations.

| Score | Description |
| --- | --- |
| 0 | No symptoms at all |
| 1 | No significant disability despites symptoms; able to carry out all usual duties and activities |
| 2 | Slight disability; unable to carry out all previous activities, but able to look after own affairs *and eat* without assistance |
| 3 | Moderate disability; requiring some help, but able to walk without assistance, *needs assistance to eat or drink water by syringe and facial paralysis* |
| 4 | Moderately severe disability; unable to walk without assistance and unable to attend to own bodily needs without assistance, *circling, pressing head along walls of pen, and standing* |
| 5 | Severe disability; bedridden, incontinent and requiring constant nursing care and attention |
| 6 | Dead |

**Supplementary Figure 1**

**
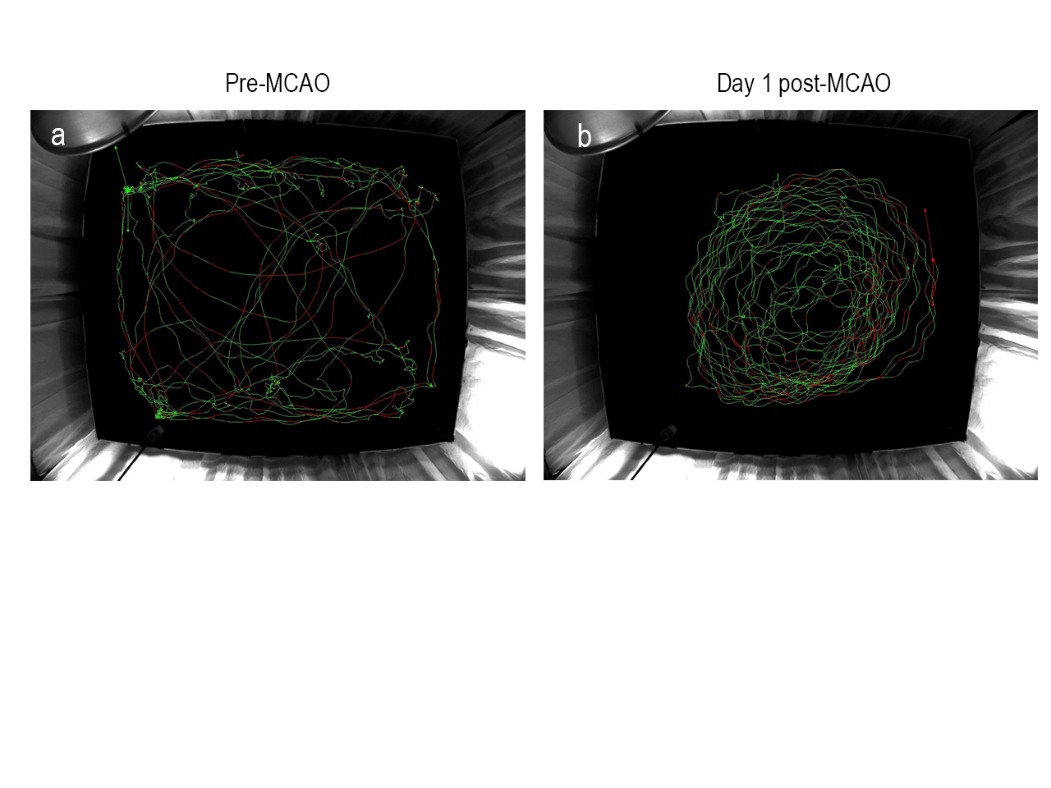
**
